# Fertilization treatments affect soil CO_2_ emission through regulating soil bacterial community composition in the semiarid Loess Plateau

**DOI:** 10.1038/s41598-022-21108-4

**Published:** 2022-11-22

**Authors:** Jinbin Wang, Junhong Xie, Lingling Li, Zechariah Effah, Lihua Xie, Zhuzhu Luo, Yongjie Zhou, Yuji Jiang

**Affiliations:** 1 State Key Laboratory of Aridland Crop Science, Gansu Agricultural University, Lanzhou, 730070 China; 2 College of Agronomy, Gansu Agricultural University, Lanzhou, 730070 China; 3 College of Resource and Environment, Gansu Agricultural University, Lanzhou, 730070 China; 4 State Key Laboratory of Soil and Sustainable Agriculture, Institute of Soil Science, Chinese Academy of Sciences, Nanjing, 210008 China

**Keywords:** Agroecology, Climate-change ecology, Microbial ecology

## Abstract

A growing body of literature have emphasized the effects of fertilization regimes on soil respiration and microbial community in the semiarid region, however, fertilization treatment effects on the soil CO_2_ emission, soil bacterial community, and their relationships from long-term experiments is lacking. In the present study, we investigated the effects of long-term fertilization regimes on soil bacterial community and thereafter on soil CO_2_ emission. A 9-year field experiment was conducted with five treatments, including no fertilizer (NA) and four fertilization treatments (inorganic fertilizer (CF), inorganic plus organic fertilizer (SC), organic fertilizer (SM), and maize straw (MS)) with equal N input as N 200 kg hm^–2^. The results indicated that CO_2_ emission was significantly increased under fertilization treatments compared to NA treatment. The bacterial abundance was higher under MS treatment than under NA treatment, while the Chao1 richness showed opposite trend. MS treatment significantly change soil bacterial community composition compared to NA treatment, the phyla (Alphaproteobacteria and Gammaproteobacteria) and potential keystone taxa (*Nitrosomonadaceae* and *Beijerinckiaceae*) were higher, while the Acidobacteriota was lower under MS treatment than under NA treatment. CO_2_ emission was positively correlated with the abundance of Alphaproteobacteria, Gammaproteobacteria, and keystone taxa, negatively correlated with these of Acidobacteriota. Random forest modeling and structural equation modeling determined soil organic carbon, total nitrogen, and the composition and network module III of the bacterial community are the main factors contribute to CO_2_ emission. In conclusion, our results suggest that the increased CO_2_ emission was affected by the varied of soil bacterial community composition derived from fertilization treatments, which was related to Alphaproteobacteria, Gammaproteobacteria, Acidobacteriota, and potential keystone taxa (Nitrosomonadaceae and Beijerinckiaceae), and highlight that the ecological importance of the bacterial community in mediating carbon cycling in the semiarid Loess Plateau.

## Introduction

Soil releases large amounts of CO_2_ to the atmosphere through respiration, which leads to an increase in atmospheric CO_2_ concentration, thus threatens ecological sustainability^1^. Agricultural system is one of the most active components of the terrestrial ecosystem and plays crucial roles in global C cycling^2^. Thus, even small changes in the agricultural system may significantly alter soil respiration, and profoundly affect the atmosphere CO_2_ concentration^3^ and global C cycling^4^. Soil microbial and plant roots respiration are the key components of soil respiration^5^; meanwhile, these two pathways are simultaneously influenced by biological and abiotic properties^6^. To date, the information is limited to the biological mechanisms of these factors regulating soil respiration.

Evidence has demonstrated that soil temperature^7^, soil moisture^8^, plants diversity^9^, and soil physicochemical properties^10^ are the important factors for predicting soil respiration. However, uncertainties are particularly high in farming ecosystems, in which small changes in climate^11^ and fertilization practices can strongly impact microbial C metabolism and cycling dynamics^12^. Soil microorganisms play significant roles in predicting CO_2_ emission through microbial processes^13^. Especially, microbial diversity plays important ecological roles in mediating multiple ecosystem functioning^14^, including soil CO_2_ emission and climate regulation. In addition to the diversity, the microbial community structures and co-occurrence network may be responsible for soil CO_2_ emission. Studies have shown that Proteobacteria and Actinobacteria are positively correlated with CO_2_ emission^7,15^, suggesting that the compositions of these microbial community have potential roles in driving CO_2_ emission. So far, how the composition and network of the microbial community changed CO_2_ emission has not been fully assessed under field studies in dryland farming system.

Maize (*Zea mays* L.) is the dominant crop for food security in the semiarid Loess Plateau of China. Fertilization is often considered to provide the limiting element for maize growth in this region. The long-term dependence on inorganic fertilizer results in a decline in soil indicators^16^ and N use efficiency^17^, while organic plus inorganic fertilizer is helpful to improve soil fertility and maize yield^18,19^. The fertilizer application also has profound impacts on soil C pools and C fluxes^5^. Although a large number of experiments have been conducted to explore the effects of fertilization on soil CO_2_ emission, it is still controversial showed positive^20^, negative^21^, or neutral effects^22^ on CO_2_ emission due to the complexity of farmland system. Therefore, understanding the effects of fertilization on CO_2_ emission is vital for predicting regional and global C cycling. Furthermore, fertilization regimes in farmland system result in a high variation in the diversity and composition of soil bacterial community^23^. Study showed that soil biomass and microbial diversity play potential roles in mediating soil CO_2_ emission^24^, while there are also studies reported that fertilization amendments regulate soil CO_2_ emission through changing soil bacterial community composition, rather than diversity and biomass^13,15^. Therefore, disentangling how fertilization treatments affect soil microbial community and its relationship with CO_2_ emission is of great significance for mediating C cycling in farmland.

Here, we aimed to explore how the bacterial community regulate the response of CO_2_ emission to fertilization treatments. In the present study, a 9-year field experiment with five treatments was performed in the semiarid Loess Plateau. Specifically, we addressed the following questions: (i) how does soil CO_2_ emission change in response to fertilization treatments? (ii) how do fertilization treatments affect the diversity, composition, and co-occurrence network of soil bacterial community? and (iii) what is the ecological mechanism of soil bacterial community in driving soil CO_2_ emission across fertilization treatments? We hypothesized that fertilization treatments significantly changed the composition and co-occurrence network of soil bacterial community through improving soil chemical properties. We expected that the bacterial community composition mediated soil CO_2_ emission under fertilization treatments.

## Results

### Soil chemical properties and CO_2_ emission

**Figure 1:**
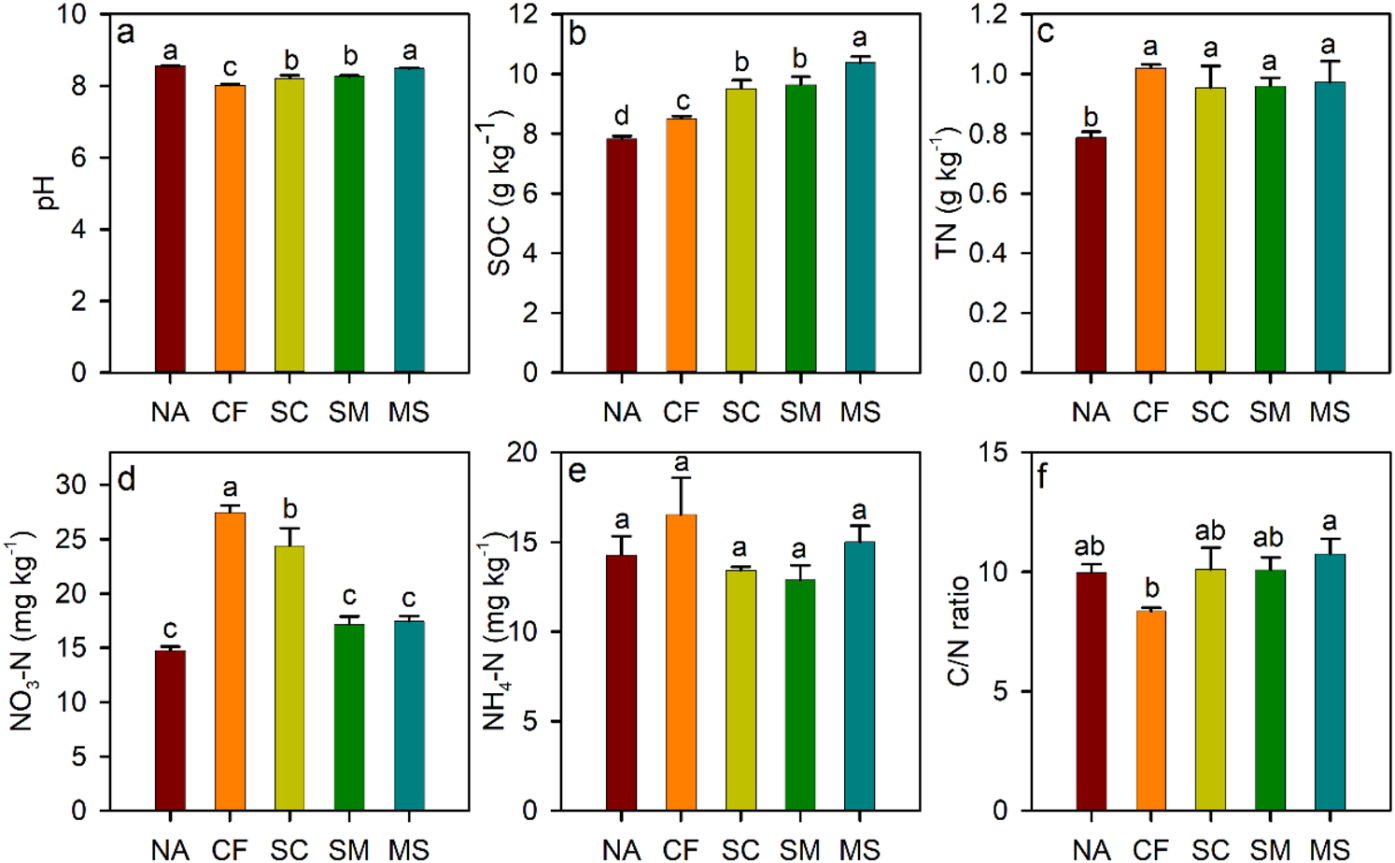
Soil properties under different treatments. SOC, soil organic carbon; TN, total nitrogen; NO_3_-N, nitrate nitrogen; NH_4_-N, ammonium nitrogen; C/N ratio, SOC to TN ratio. NA, no fertilizer; CF, inorganic fertilizer; SC, inorganic fertilizer plus organic fertilizer; SM, organic fertilizer; MS, maize straw. Different letters indicate significant difference at *p* < 0.05.

**Figure 2:**
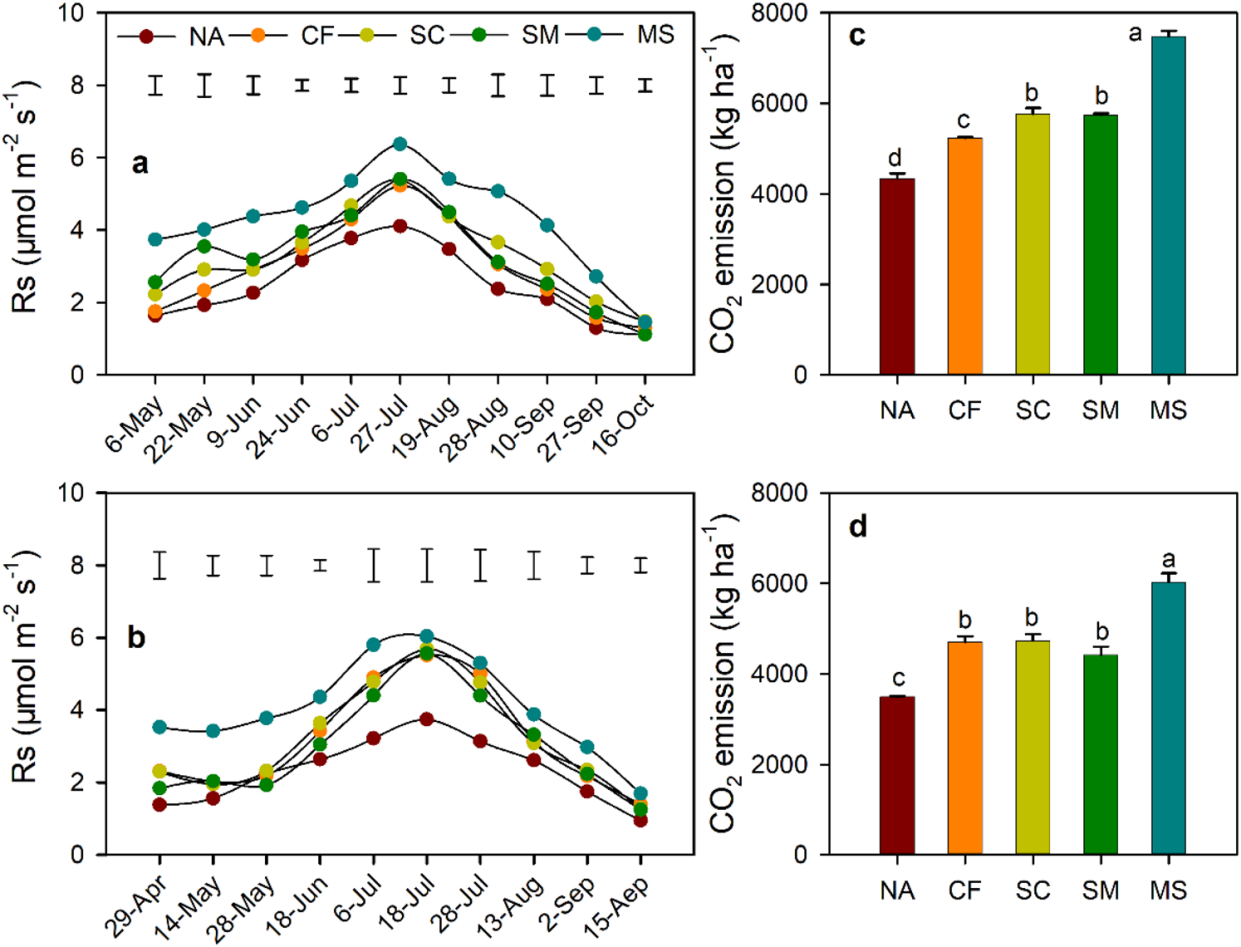
Soil respiration (Rs) and CO_2_ emission in 2020 (**a** and **c**) and 2021 (**b** and **d**) under different treatments. NA, no fertilizer; CF, inorganic fertilizer; SC, inorganic fertilizer plus organic fertilizer; SM, organic fertilizer; MS, maize straw. Bars in figure (**a** and **b**) represent LSD values (*p* < 0.05). Different letters in figure (**c** and **d**) indicate significant difference at *p* < 0.05.

Soil pH, SOC, TN, AP, C/N, and total CO_2_ emission obey normality, while NO_3_-N do not obey normality. Soil pH was significantly lower under CF, SC, and SM treatments than under NA treatment (Fig. 1). SOC was significantly higher under MS treatment than under CF and SC treatments, while NO_3_-N exhibited the inverse trend. The C/N ratio under MS treatment was significantly improved compared to CF treatment. No significant difference in TN and NH_4_–N was observed among fertilization treatments. Soil respiration exhibited similar variations during the growing season of maize (Fig. 2a,b). Compared to NA treatment, the mean Rs and total CO_2_ emission (TCE) increased by 28.8–74.5% and 27.1–72.7% under fertilization treatments in both years, respectively (Fig. 2c,d). Furthermore, the mean values of Rs and TCE were significantly enhanced under MS treatment than under CF, SC, and SM treatments.

### Soil bacterial community

Both the abundance, Chao1 richness, and Shannon index of soil bacterial community obey normality. The abundance of soil bacteria represented by the copy number of 16S rRNA gene ranged from 0.57 × 10^7^ to 1.14 × 10^7^ copies g^−1^ soil (Fig. S1). The bacterial abundance was significantly higher under MS treatment than under NA and CF treatments. The Chao1 richness was significantly increased under SM treatment, but decreased under MS treatment compared to NA treatment. No significant differences were observed in Shannon index among five treatments.

**Figure 3:**
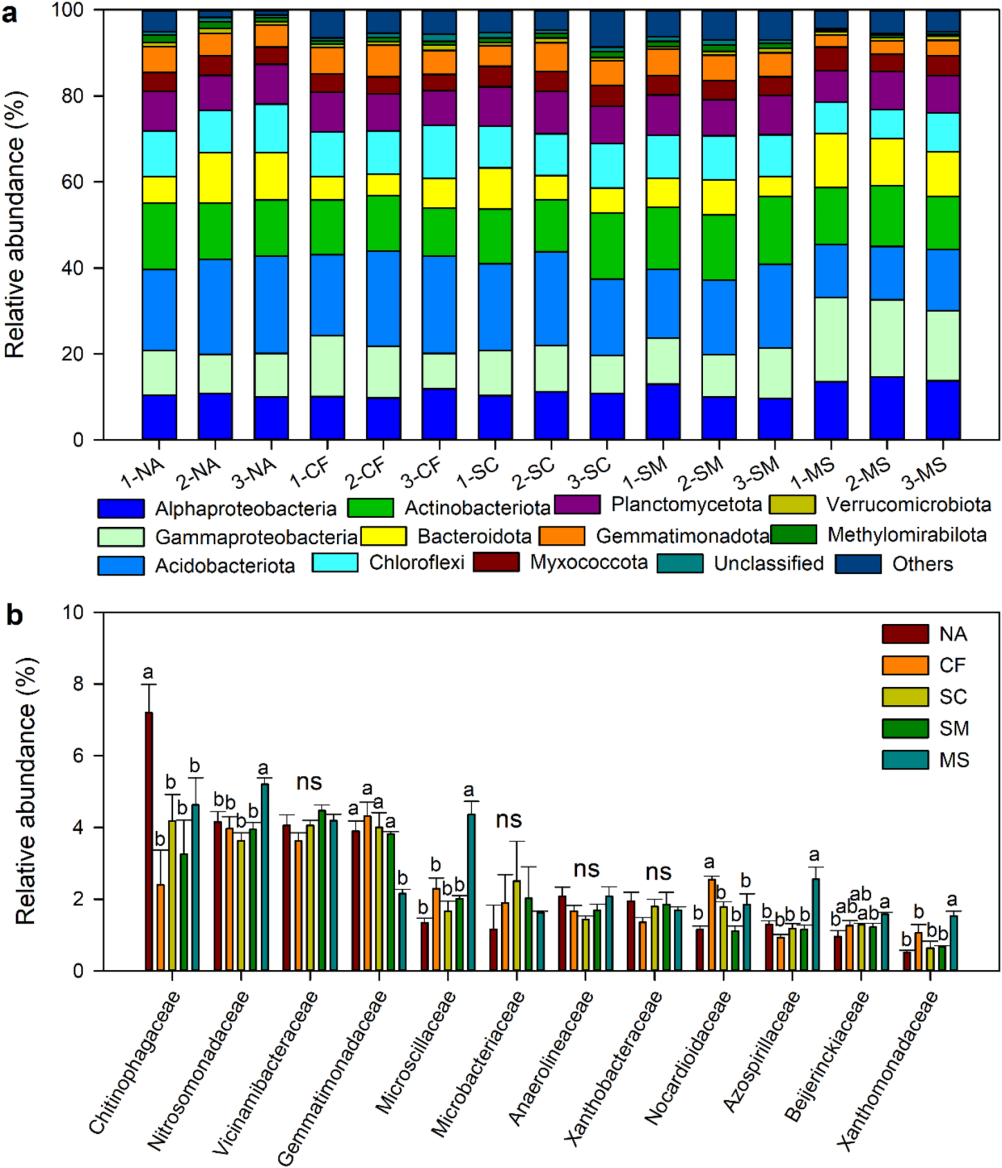
Changes in bacterial community at phylum level (**a**). The numbers before the treatment name indicate the sampling replications. (**b**) The bacterial community at family level. Different letters indicate significant difference at *p* < 0.05. ns represent no significant differences among treatments. NA, no fertilizer; CF, inorganic fertilizer; SC, inorganic fertilizer plus organic fertilizer; SM, organic fertilizer; MS, maize straw.

**Figure 4:**
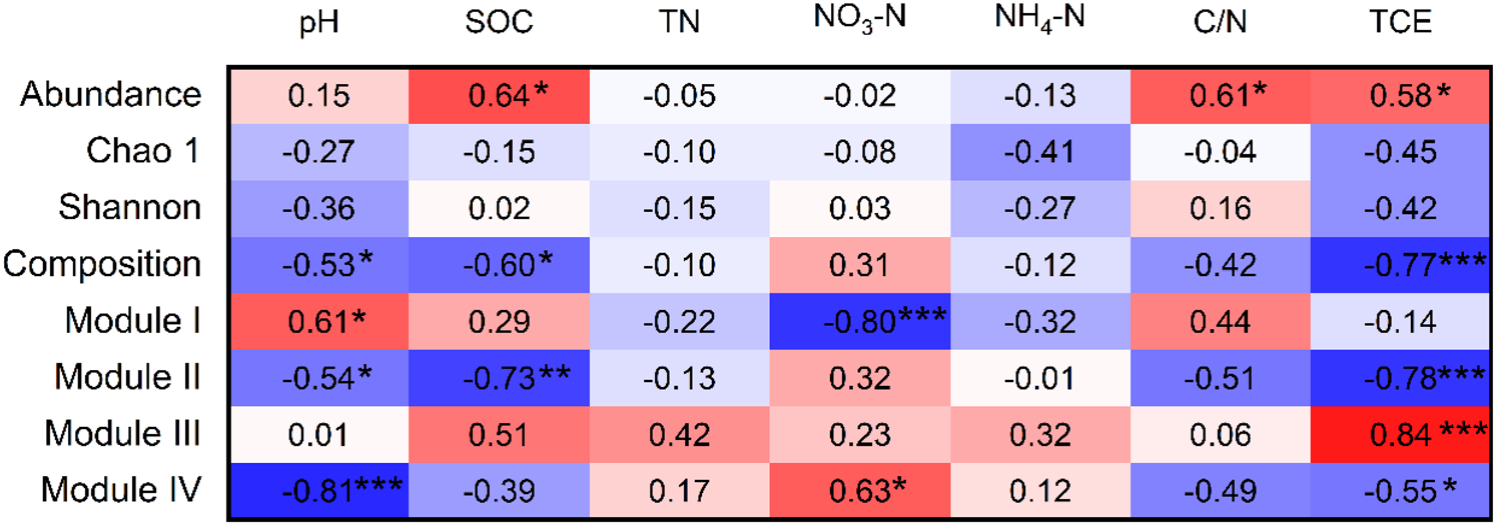
The relationships between the bacterial community, soil properties, and CO_2_ emission. SOC, soil organic carbon; TN, total nitrogen; NO_3_-N, nitrate nitrogen; NH_4_-N, ammonium nitrogen, C/N ratio, SOC to TN ratio, TCE, total CO_2_ emission. *** *p* < 0.001; ** *p* < 0.01; * *p* < 0.05.

The bacterial community compositions were mainly consisted of Alphaproteobacteria (11.3%), Gammaproteobacteria (12.0%), Acidobacteriota (17.7%), Actinobacteriota (13.6%), (Fig. 3a). The abundance of Alphaproteobacteria and Gammaproteobacteria were significantly increased, but that of Acidobacteriota was decreased under MS treatment compared with the other treatments. The dominant families in the bacterial community were *Chitinophagaceae* (4.3%), *Nitrosomonadaceae* (4.2%), *Vicinamibacteraceae* (4.1%), and *Gemmatimonadaceae* (3.6%) (Fig. 3b). The abundance of *Chitinophagaceae* was significantly decreased under CF, SC, SM, and MS treatments compared to NA treatment. The abundance of *Nitrosomonadaceae* and *Beijerinckiaceae* were significantly higher under MS treatment than under NA, CF, SC, SM treatments, while *Gemmatimonadaceae* exhibited opposite trend (Fig. 3b). Principal coordinate analysis indicated that the bacterial community composition was significantly affected by fertilization treatments (Fig. S2). The bacterial abundance was positively correlated with SOC, C/N ratio, and TCE, while the bacterial community composition was negatively correlated with pH, SOC, and TCE (Fig. 4). TCE was positively correlated with the abundance of Alphaproteobacteria, Gammaproteobacteria, but negatively correlated with these of Acidobacteriota (Table S1).

### Co-occurrence networks of soil bacterial community

**Figure 5:**
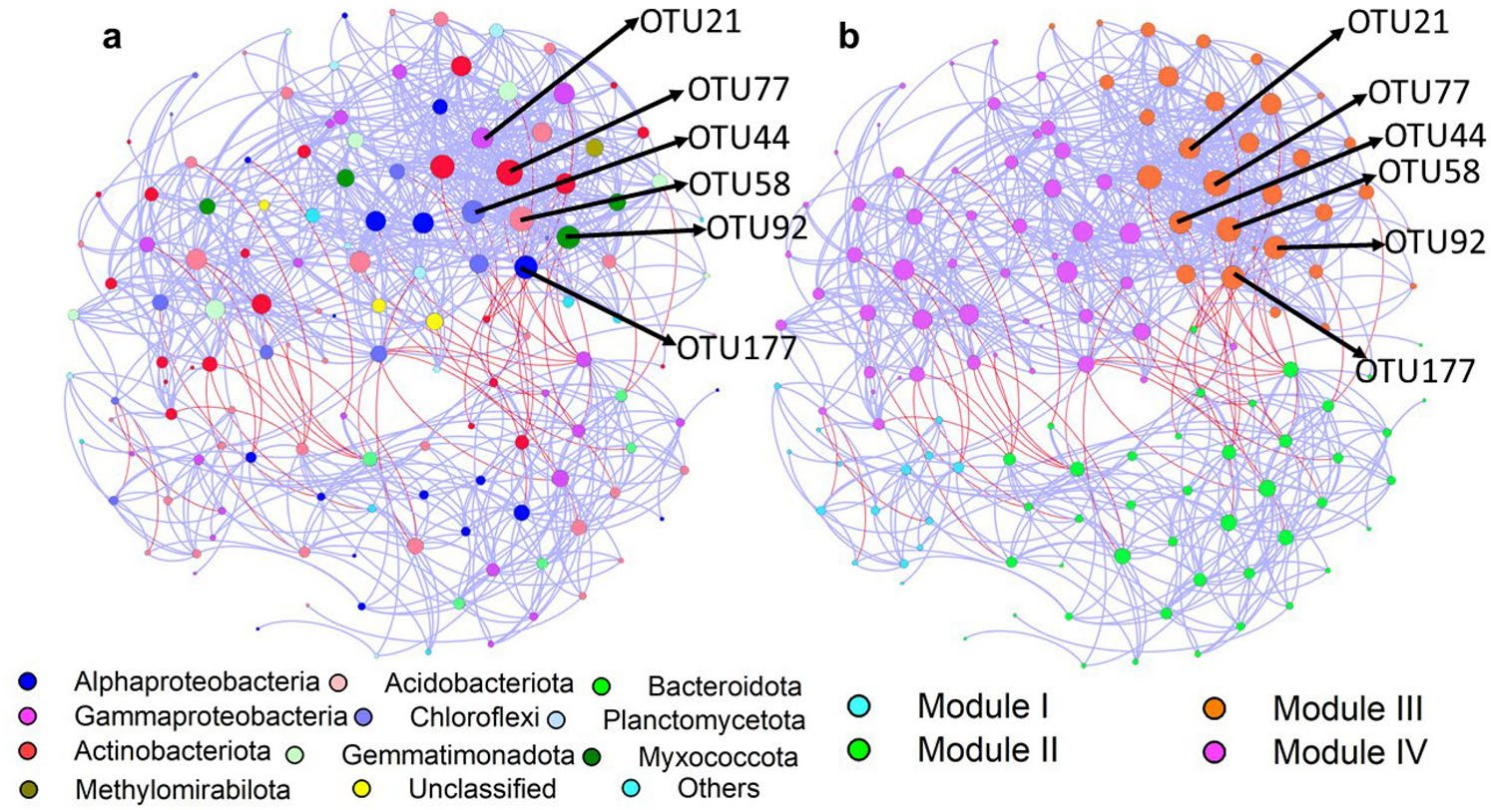
Interaction networks of soil bacterial community based on different N treatments. The networks are colored by phylum (**a**) and module (**b**), respectively. Modules I, II, III, and IV represent four clusters with tightly linked nodes. Size of each node is proportional to the number of connections (i.e., degree), and the thickness of each connection between two nodes (i.e., edge) is proportional to the value of Spearman’s correlation coefficients. Blue edges indicate positive interactions, while red edges indicate negative connections. The OTUs shown in networks are keystone taxa.

To reveal the co-occurrence pattern of soil bacterial community in five treatments, a network was constructed based on 15 samples (Fig. 5). There were 140 nodes, 798 edges, and 4 modules in the networks. The bacterial network had more positive edges (750) than negative edges (48) (Table S2). In particular, the statistical keystone taxa belonged to *Nitrosomonadaceae*, *Vicinamibacteraceae*, and *Beijerinckiaceae* (Table S3), and the edges associated with potential keystone taxa were mostly positive in module III. Modules I and IV were significantly correlated with pH and NO_3_-N, Module II was negatively correlated with pH and SOC. Module II and IV had negatively correlations with TCE, Module III had positive correlation with TCE (Fig. 4). In addition, TCE was positively correlated with the relative abundance of *Nitrosomonadaceae* and *Beijerinckiaceae* (Fig. S3).

### Soil properties and bacterial community regulated CO_2_ emission

**Figure 6:**
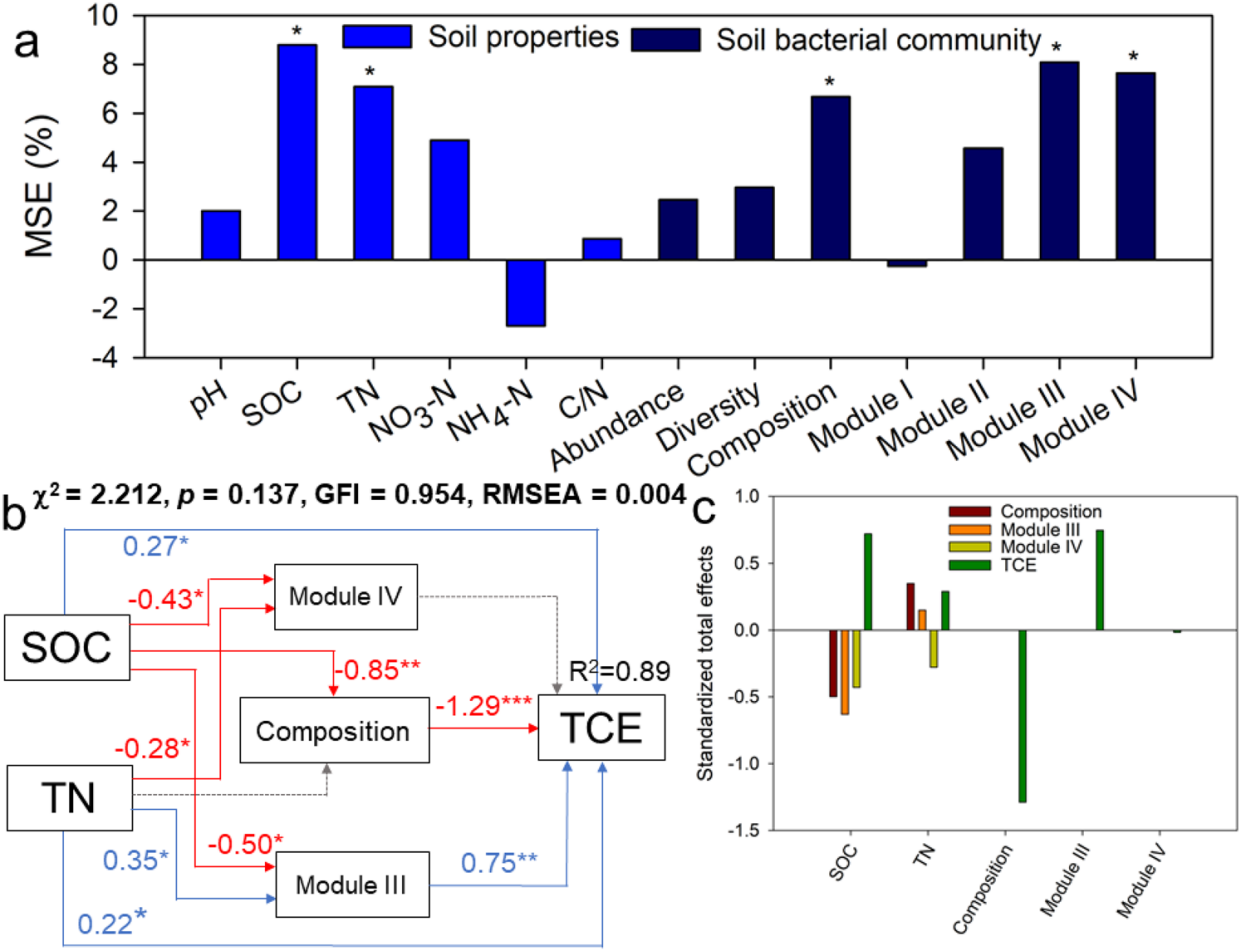
The significant predictors of soil properties and the bacterial community to total carbon emission (TCE) via random forest analysis (**a**). The bacterial community is represented by abundance (copy number of 16S rRNA), diversity (Shannon index), composition (the first principal coordinate), and module III and VI (module eigengenes) of the bacterial networks. **(b)** Effects of soil properties and the bacterial community on TCE as estimated using structural equation modeling (SEM) analysis. Blue and red lines indicate significant positive and negative relationships, respectively. (**c**), The standardized total effects for the indexes in the SEM. TN, total nitrogen; SOC, soil organic carbon; NO_3_-N, nitrate nitrogen; NH_4_-N., ammonia nitrogen, C/N ratio, SOC to TN ratio. *** *p* < 0.001; ** *p* < 0.01; * *p* < 0.05.

Random forest modeling revealed that SOC and TN were the main soil properties for predicting CO_2_ emission (Fig. 6a). As for the bacteria community, the bacterial compositions, and modules III and VI in the bacterial network significantly affected soil CO_2_ emission. Structural equation modeling (SEM) further suggested that SOC and TN had significant correlations with the composition and network (modules III and IV) of the bacterial community (Fig. 6b). Importantly, CO_2_ emission was negatively associated with the bacterial community composition (*p* < 0.001), but positively correlated with the module III of bacterial network (*p* < 0.05).

## Discussion

### Fertilization treatments affected soil CO_2_ emission and the bacterial community

In the semiarid Loess Plateau, nitrogen fertilization is often regarding as the common practice to improve soil quality and crop yield due to where there is a low N level^25^. However, fertilization regimes also significantly influence soil CO_2_ emission^26^. The effect of fertilization treatments on CO_2_ emission was related the autotrophic (Ra) and heterotrophic respiration (Rh), which was significantly affected by crop growth and soil substrate^27^. In the present study, soil CO_2_ emission significantly increased by 27.1–72.7% under fertilization treatments compared to the NA treatment. This result was consistent with the results from previous studies that reported increased CO_2_ emission after fertilization in farmland ecosystems^28,29^. The CF treatment promoted CO_2_ emission could attributed to the synchrony between nutrient supply and crop nutrient demand, and enhance maize growth, leave area index, biomass, promoted the allocation of carbohydrates belowground and maize roots activity, thus resulting in the increase CO_2_ emission through Ra^28^. The increased CO_2_ emission under SC, SM, and MS treatments mainly because the high level of SOC, which supply higher concentrations of nutrient substrates for the microbial community, thus stimulated soil heterotrophic respiration and CO_2_ emission^30^. Moreover, CO_2_ production from SOC mineralization also significantly contributes to total CO_2_ emission^31^. CO_2_ emission was significantly higher under MS treatment than under other treatments may because the high SOC mineralization in early stage of maize, which would increase soil respiration.

In this study, organic treatments (SC, SM, and MS) increased the bacterial abundance compared to the NA and CF treatments. The result was in accordance with previous researches^32,33^. The large bacterial abundance was probably explained by high SOC and appropriate C/N under organic treatments. Soil C/N ratio has also been considered to play crucial roles in enhancing soil bacterial abundance^34^. The appropriate C/N supply a favorable habitat for the bacterial community and the readily available C substrates can serve as food resources for the bacterial growth^35,36^. In addition, the bacterial abundance under MS treatment was significantly higher than under NA treatment may attribute to more maize straw could help some soil-dominant bacteria proliferation, like Proteobacteria^37^. We found that the Chao1 richness was significantly decreased under MS treatment, but increased under SM treatment compared to the NA treatment. Previous studies have reported that the application of maize straw shows stimulatory effects on bacterial phyla Proteobacteria and Firmicutes, and fungal ascomycetes^38^, and reduces species richness due to the disappearance of locally rare species^39^. In contrast, soil bacteria can take full advantage of nutrients in organic fertilizer and thus improve the bacterial Chao1 richness^40^.

Soil bacterial community composition was significantly varied by fertilization treatments, and significantly correlated with soil pH and SOC, which was consistent with the findings of previous studies^41,42^. Soil pH plays a key role in regulating soil bacterial community^41,43^, and that high SOC dominated by the fresh labile organic C has significant effects on the composition of the bacterial community^44^. Therefore, the difference in soil bacterial community composition could be explained by significant changes in pH and SOC across the five treatments. The bacterial communities were mainly assigned to Alphaproteobacteria, Gammaproteobacteria, Acidobacteriota, Actinobacteriota. All these bacteria are previously reported that have specific roles in utilizing SOC for respiration^13,15^. The MS treatment exhibited the highest relative abundance of Alphaproteobacteria and Gammaproteobacteria, suggesting that a high SOC level is beneficial to growth and colonization of these two classes. The phylum Proteobacteria has generally been categorized as copiotrophs, preferring nutrients-rich environments^15,45^. The phylum Acidobacteriota is significantly correlated with soil pH and N availability^46,47^. Thus, the high abundance of the phylum Acidobacteriota under CF and SC treatments may be explained by the fact that the pH was closer to neutral and increased NO_3_-N.

Co-occurrence network is a crucial tool to assess the function of soil microbial community, which reveals the potential connections and ecological niches structure among bacterial taxa in soil^48,49^. In this study, the high edges between nodes in the bacterial network suggested the close relationships between bacterial taxa. The underlying connections among the taxa in bacterial network indicate the exchanging nutrients and metabolic products^50^. The high positive correlations in the bacterial network represented potential commensalism relationships existing between the bacterial taxa^51^. In addition, each module group represent the clustering of species may share the same ecological niches, and have similar responses to environmental changes^52^. Moreover, the modules in networks have been determined to originate from interaction specificity, habitat heterogeneity, resource partition, and ecological niche overlap^53^, and optimize ecological functions of nutrients cycling and resources availability in the farmland system^51,54^. Furthermore, we discovered that *Nitrosomonadaceae*, *Vicinamibacteraceae*, and *Beijerinckiaceae* were the presumed keystone taxa. The keystone taxa play strong roles in maintaining potential biochemical potential functions of soil microbial community^55,56^.

### Soil bacterial community regulated CO_2_ emission

Soil bacterial community have been reported as new insights in regulating soil respiration and CO_2_ emission^13^. Soil microbial respiration is a key component of soil respiration, and is positively correlated with soil microbial biomass^57^. The present study showed that soil CO_2_ emission was positively correlated with soil bacterial abundance, suggesting that increasing microbial populations may significantly contribute to soil CO_2_ emission, this result was consistent with previous study^57^. Although soil bacterial diversity plays important roles in regulating soil basic respiration^58^, our study found no relationship between soil CO_2_ emission and bacterial diversity. It has been documented that soil respiration dynamics is determined by a certain part of taxa in the bacterial composition, rather than over diversity^59^. Therefore, a better understanding of soil respiration is required to incorporate associations in the bacterial community. We found that soil bacterial community composition had a negative correlation with soil CO_2_ emission, which was in agreement with Liu et al.^13^ and Chen et al.^15^, who reported that the bacterial community composition may play crucial roles in regulating soil CO_2_ emission. It has been broadly reported that copiotrophs and oligotrophs have the ability of utilizing C for respiration. Generally, oligotrophs are proposed to exhibit lower respiration rates than copiotrophs^13,60,61^. The Alphaproteobacteria and Gammaproteobacteria are considered as potential copiotrophs, whereas Actinobacteria are classified as oligotrophs^60^. In the present study, the relative abundance of Alphaproteobacteria and Gammaproteobacteria was significantly improved under MS treatment than under other treatments, while that of Actinobacteria followed a decreasing pattern. Consequently, the activity of bacterial populations with the changes in oligotroph-to-copiotroph ratio within the community may be accounted for the negative relationship between the bacterial community composition and CO_2_ emission across fertilization treatments. Furthermore, we found there were remarkable differences among the four distinct modules in the network, and the module III in network exerted a strong positive relationship with soil CO_2_ emission. Meanwhile, the keystone taxa were all in module III of the network. The potential keystone taxa in the soil microbial community are identified to exert significant contributions to SOC sequestration and carbon cycling dynamics irrespective of their abundance^62,63^. In the present study, the keystone taxa *Nitrosomonadaceae* and *Beijerinckiaceae* had positive correlations with soil CO_2_ emission. Studies have revealed that these two dominant taxa occupy specific ability soil C for respiration. The family *Beijerinckiaceae* is positively correlated with decomposition rates of organic matter^64^, *Nitrosomonadaceae* promote soil C mineralization^65^, and eventually increased soil CO_2_ emission. Thus, soil bacterial community shift with high abundance of *Nitrosomonadaceae* and *Beijerinckiaceae* may considerably improve soil respiration and CO_2_ emission.

Taken together, our results highlight that the increased CO_2_ emission was affected by the varied of soil bacterial community composition derived from fertilization treatments, which was related to Alphaproteobacteria, Gammaproteobacteria, Acidobacteriota in the semiarid Loess Plateau. We further provide new insights into the potential underlying mechanisms of keystone taxa (*Nitrosomonadaceae* and *Beijerinckiaceae*) mediating CO_2_ emission in farmland systems through farmland management practices. However, it is worth noting that the associations between soil bacterial community composition and CO_2_ emission based on statistical correlations do not necessarily represent causal relationships. Future research using stable isotope tracking is warranted to verify the casual links between soil bacterial community and CO_2_ emission.

## Conclusion

Our results indicated that fertilization treatments significantly increased CO_2_ emission compared to the NA treatment. The bacterial abundance was higher, while the Chao1 richness was lower under MS than under NA treatment. Fertilization treatments significantly affected the Alphaproteobacteria, Gammaproteobacteria, Acidobacteriota, thus changed soil bacterial community composition. Soil organic carbon, total nitrogen, and the community composition and network module III of soil bacterial community contributed to CO_2_ emission. We further identified CO_2_ emission was positively correlated with the potential keystone taxa (Nitrosomonadaceae and Beijerinckiaceae). Overall, this study provided the increased CO_2_ emission was affected by the varied of soil bacterial community composition derived from fertilization treatments, which was related to Alphaproteobacteria, Gammaproteobacteria, Acidobacteriota, and potential keystone taxa (*Nitrosomonadaceae* and *Beijerinckiaceae*), and may highlight that the ecological importance of the bacterial community in mediating carbon cycling in the semiarid Loess Plateau.

## Materials and methods

### Site description and experimental design

The filed study was located at the Rainfed Agricultural Experimental Station (35°28’N, 104°44’E) of Gansu Agricultural University in Gansu province, China. Long-term mean annual temperature and precipitation of the area were 6.4 °C and 390 mm, respectively. The soil at the experimental site is described as Calcaric Cambisol according to the Food and Agriculture Organization classification system. The field experiment with five treatments was started in 2012, including no fertilizer (NA), inorganic fertilizer (CF), inorganic plus organic fertilizer (SC), organic fertilizer (SM), and maize straw (MS). Each treatment had three replicates and 15 plots (13 m length and 3.3 m width) were formed based on a randomized block design. The CF, SC, SM, and MS treatments received the same rate (200 kg N ha^−1^) of N fertilizers. Chemical N fertilizers were applied as urea (46% N). Organic fertilizer was made from cow manure, which contained 3.3% of N, 1.0% of P_2_O, 0.7% of K_2_O_5_, respectively. Maize straw content of N, P_2_O, K_2_O_5_ were 0.7%, 0.4%, and 0.5%, respectively. All fertilizers were evenly applied 10 days prior to sowing. Maize variety (cv. Pioneer 335) was planted at 52,500 plants ha^−1^ in late April and harvest in early October.

### Sampling and soil properties assays

In August 2021, nine soil samples were collected from topsoil depth (0–20 cm) in each plot following an S-shaped sampling strategy, and then mixed to form a composite sample. Visible residues and roots were removed using a 2-mm soil sieve. The samples were then separated into two parts. One part was immediately stored at − 80 °C for soil bacterial community’ analysis, and the other part was air-dried for chemical analysis. Six soil properties were determined, including soil pH, soil organic C (SOC), total N (TN), nitrate N (NO_3_-N), ammonium N (NH_4_-N), and the ratio of SOC to TN (C/N ratio). Detailed soil chemical analysis was done as described by Bao^66^.

### Soil respiration rate and CO_2_ emission

After crop seeding, three PVC collars were randomly pressed into each plot to measure soil respiration (Rs). The Rs rate was determined using a Li-8100A automated soil CO_2_ flux system from 09:00 am to 11: 00 am with about 15-day intervals throughout the growing season from May 6 to October 16 in 2020, and April 29 to September 15 in 2021, respectively. Total CO_2_ emission (TCE) across the maize growing season was calculated according to the following equation^67^.

1 $$\mathrm{TCE}=\sum \left[\frac{\mathrm{Rs}\left(\mathrm{i}+1\right)+\mathrm{Rs}(\mathrm{i})}{2}\times \left[\mathrm{t}\left(\mathrm{i}+\mathrm{i}\right)-\mathrm{t}(\mathrm{i})\right]\times 0.1584\times 24\right]\times 0.2727\times 10$$

where i + 1 is current measuring date, i is previous measuring date, and t is days after seeding.

### Soil bacterial community

Soil DNA was extracted from 0.5 g fresh samples using the EZNA® Soil DNA Kit. The extracted DNA concentration and purity were measured using a NanoDrop-2000 spectrophotometer (Thermo Fisher Scientific, Wilmington, DE, USA). The abundance of bacteria was evaluated by quantifying the copy number of 16S rRNA gene using the primers 515F and 907R^68^. Standard curves were obtained using tenfold serial dilutions of plasmids DNA containing 16S rRNA gene. The amplification efficiency and correlation coefficients for the standard curve were 97.0% and 0.99, respectively. The bacterial abundance was calculated based on the standard curves.

High-throughput sequences of PCR products were obtained from the Illumina MiSeq platform. Raw sequences were trimmed and quality-filtered using the QIIME software (v1.8.0) to obtain clean paired-end reads. FLASH (version 1.2.11) software was used to merge the clean paired-end reads and used MOTHUR software (V1.35.1) to remove the barcode and primers. Operational taxonomic units (OTUs) were clustered at a similarity level of 97% by the UPARSE (v7.1) software package. Bacterial sequences were taxonomically identified based on SILVA database^69^. The bacterial diversity (Shannon index and Chao1 richness) was calculated using MOTHUR software after comparing all samples. A total of 31,346 bacterial OTUs were obtained across all samples. The sequences of bacteria have been deposited in the NCBI database (Accession number: PRJNA821401).

### Statistical analysis

All data were checked for normality by the Shapiro–Wilk test using SPSS 22.0 (IBM SPSS, USA), and for homoscedasticity using Levene’s test with the general linear model. Either square root or natural log transformation was used to transform data to achieve normality when assumptions could not be met. One-way ANOVA with LSD testing (*p* < 0.05) was used to evaluate the significant differences in soil properties, CO_2_ emission, and the bacterial community among the five treatments. The relationships between soil properties, soil bacterial community, and CO_2_ emission were assessed by Pearson’s correlation coefficients analysis. Principal coordinate analysis (PCoA) was performed to explore the Bray–Curtis distances of soil bacterial community under the five treatments. The permutational multivariate analysis of variance (PERMANOVE) was used to test the significance of the principal coordinate analysis.

Co-occurrence networks were constructed to visualize the relationships among bacterial community. 15 samples were mixed together and OTUs with a number of more than 10 were selected for network analysis. Correlations between the bacterial taxa were calculated by Spearman’s correlation. A true co-occurrence was considered a statistically robust correlation between species when the Spearman’s correlation coefficient was > 0.6 or <  −0.6 and the *P* < 0.05. To reduce the chances of obtaining false-positive results, Benjaminie-Hochberg method was used to test the correlations^70^. Gephi software was used to visualize the co-occurrence network and soil taxa were categorized as four distinct modules closely associated nodes^71^. OTUs with high degree, high eigenvector centrality, and high closeness/betweenness centrality were considered as the keystone taxa^48^.

The main predictors of CO_2_ emission were estimated using random forest analysis. The ‘randomForest’ package was used to investigate random forest modeling^72^. The significance of model predictors was determined using the package ‘rfPermute’^73^. The significant predictors from random forest analysis were further selected for structural equation modeling (SEM) analysis. SEM analysis was applied to determine the direct and indirect contributions of soil properties and the bacterial community to CO_2_ emission. SEM analysis was performed using AMOS 22.0 software (SPSS, Chicago, IL, USA). The model fitness was evaluated by χ^2^ (*p* > 0.05), goodness-of-fit, and root mean square error of approximation^74^.

## Supplementary Information

Supplementary Information.

## Data Availability

The datasets presented in this study can be found in online repositories. The names of the repository/repositories and accession number(s) can be found at: https://www.ncbi.nlm.nih.gov/bioproject/PRJNA821401.

## References

[1] Bond-Lamberty B, Thomson A (2010). Temperature-associated increases in the global soil respiration record. Nature.

[2] Crippa M (2021). Food systems are responsible for a third of global anthropogenic GHG emissions. Nat. Food.

[3] Shakoor A (2021). Effect of animal manure, crop type, climate zone, and soil attributes on greenhouse gas emissions from agricultural soils—A global meta-analysis. J. Clean Prod..

[4] Wu L (2019). Soil organic matter priming and carbon balance after straw addition is regulated by long-term fertilization. Soil Biol. Biochem..

[5] Chen F (2019). Effects of N addition and precipitation reduction on soil respiration and its components in a temperate forest. Agr. Forest. Meteorol..

[6] Lei J (2021). Temporal changes in global soil respiration since **1987**. Nat. Commun..

[7] Wang R (2019). Nitrogen application increases soil respiration but decreases temperature sensitivity: Combined effects of crop and soil properties in a semiarid agroecosystem. Geoderma.

[8] Du K (2021). Influence of no-tillage and precipitation pulse on continuous soil respiration of summer maize affected by soil water in the North China Plain. Sci. Total Environ..

[9] Chen X, Chen HY (2019). Plant diversity loss reduces soil respiration across terrestrial ecosystems. Global Change Biol..

[10] Lang AK, Jevon FV, Ayres MP, Matthes JH (2020). Higher soil respiration rate beneath arbuscular mycorrhizal trees in a northern hardwood forest is driven by associated soil properties. Ecosystems.

[11] Huang N (2020). Spatial and temporal variations in global soil respiration and their relationships with climate and land cover. Sci. Adv..

[12] Xiao H (2021). The regulatory effects of biotic and abiotic factors on soil respiration under different land-use types. Ecol. Indic..

[13] Liu Y-R (2018). New insights into the role of microbial community composition in driving soil respiration rates. Soil Biol. Biochem..

[14] Wagg C, Schlaeppi K, Banerjee S, Kuramae EE, van der Heijden MG (2019). Fungal-bacterial diversity and microbiome complexity predict ecosystem functioning. Nat. Commun..

[15] Chen L-F (2021). Linkages between soil respiration and microbial communities following afforestation of alpine grasslands in the northeastern Tibetan Plateau. Appl. Soil Ecol..

[16] Choudhary M (2018). Long-term effects of organic manure and inorganic fertilization on sustainability and chemical soil quality indicators of soybean-wheat cropping system in the Indian mid-Himalayas. Agr. Ecosyst. Environ..

[17] Zhang M (2018). Increasing yield and N use efficiency with organic fertilizer in Chinese intensive rice cropping systems. Field Crop. Res..

[18] Bonanomi G (2020). Repeated applications of organic amendments promote beneficial microbiota, improve soil fertility and increase crop yield. Appl. Soil Ecol..

[19] Gai X (2018). Long-term benefits of combining chemical fertilizer and manure applications on crop yields and soil carbon and nitrogen stocks in North China Plain. Agr. Water Manage..

[20] Lai R (2017). Manure fertilization increases soil respiration and creates a negative carbon budget in a Mediterranean maize (Zea mays L.)-based cropping system. Catena.

[21] Yan T (2018). Negative effect of nitrogen addition on soil respiration dependent on stand age: Evidence from a 7-year field study of larch plantations in northern China. Agr. Forest Meteorol..

[22] Peng Q (2011). Effects of nitrogen fertilization on soil respiration in temperate grassland in Inner Mongolia. China. Environ. Earth Sci..

[23] Zeng J (2016). Nitrogen fertilization directly affects soil bacterial diversity and indirectly affects bacterial community composition. Soil Biol. Biochem..

[24] Levine UY, Teal TK, Robertson GP, Schmidt TM (2011). Agriculture's impact on microbial diversity and associated fluxes of carbon dioxide and methane. ISME J..

[25] Chen Q, Liu Z, Zhou J, Xu X, Zhu Y (2021). Long-term straw mulching with nitrogen fertilization increases nutrient and microbial determinants of soil quality in a maize–wheat rotation on China's Loess Plateau. Sci. Total. Environ..

[26] Wang J (2021). The impact of fertilizer amendments on soil autotrophic bacteria and carbon emissions in maize field on the semiarid Loess Plateau. Front. Microbiol..

[27] Subke JA, Inglima I, Francesca Cotrufo M (2006). Trends and methodological impacts in soil CO_2_ efflux partitioning: a metaanalytical review. Global Change Biol..

[28] Yan W, Zhong Y, Liu J, Shangguan Z (2021). Response of soil respiration to nitrogen fertilization: Evidence from a 6-year field study of croplands. Geoderma.

[29] Lamptey S, Xie J, Li L, Coulter JA, Jagadabhi PS (2019). Influence of organic amendment on soil respiration and maize productivity in a semi-arid environment. Agronomy.

[30] Chen Z (2018). Nitrogen fertilization stimulated soil heterotrophic but not autotrophic respiration in cropland soils: A greater role of organic over inorganic fertilizer. Soil Biol. Biochem..

[31] Zheng J, Zhang X, Li L, Zhang P, Pan G (2007). Effect of long-term fertilization on C mineralization and production of CH_4_ and CO_2_ under anaerobic incubation from bulk samples and particle size fractions of a typical paddy soil. Agr. Ecosyst. Environ..

[32] Shen J, Zhang L, Guo J, Ray J, He J (2010). Impact of long-term fertilization practices on the abundance and composition of soil bacterial communities in Northeast China. Appl. Soil Ecol..

[33] Chen Q, An X, Zheng B, Ma Y, Su J (2018). Long-term organic fertilization increased antibiotic resistome in phyllosphere of maize. Sci. Total. Environ..

[34] Zhang W, Yu C, Wang X, Hai L (2020). Increased abundance of nitrogen transforming bacteria by higher C/N ratio reduces the total losses of N and C in chicken manure and corn stover mix composting. Bioresource Technol..

[35] Chen X (2020). Microbial carbon use efficiency, biomass turnover, and necromass accumulation in paddy soil depending on fertilization. Agr. Ecosyst. Environ..

[36] Wang J (2022). Nitrogen application increases soil microbial carbon fixation and maize productivity on the semiarid Loess Plateau. Plant Soil.

[37] Li J (2022). The more straw we deep-bury, the more soil TOC will be accumulated: When soil bacteria abundance keeps growing. J. Soil Sediment.

[38] Siczek A, Frąc M, Wielbo J, Kidaj D (2018). Benefits of flavonoids and straw mulch application on soil microbial activity in pea rhizosphere. Int. J. Environ. Sci. Te..

[39] Zhao S (2019). Change in straw decomposition rate and soil microbial community composition after straw addition in different long-term fertilization soils. Appl. Soil Ecol..

[40] Zhang S (2020). Cow manure application effectively regulates the soil bacterial community in tea plantation. BMC Microbiol..

[41] Jiang Y (2016). Crop rotations alter bacterial and fungal diversity in paddy soils across East Asia. Soil Biol. Biochem..

[42] Drenovsky R, Vo D, Graham K, Scow K (2004). Soil water content and organic carbon availability are major determinants of soil microbial community composition. Microb. Ecol..

[43] Rath KM, Fierer N, Murphy DV, Rousk J (2019). Linking bacterial community composition to soil salinity along environmental gradients. ISME J..

[44] Zhao F (2018). Changes of the organic carbon content and stability of soil aggregates affected by soil bacterial community after afforestation. CATENA.

[45] Goldfarb KC (2011). Differential growth responses of soil bacterial taxa to carbon substrates of varying chemical recalcitrance. Front. Microbiol..

[46] Zhao J (2021). Response of soil microbial community to vegetation reconstruction modes in mining areas of the Loess Plateau, China. Front. Microbiol..

[47] Zhang Y, Shen H (2017). Fertilization shapes bacterial community structure by alteration of soil pH. Front. Microbiol..

[48] Wang X (2021). Organic amendments drive shifts in microbial community structure and keystone taxa which increase C mineralization across aggregate size classes. Soil Biol. Biochem..

[49] Lin Y (2019). Long-term manure application increases soil organic matter and aggregation, and alters microbial community structure and keystone taxa. Soil Biol. Biochem..

[50] Woyke T (2006). Symbiosis insights through metagenomic analysis of a microbial consortium. Nature.

[51] Zhang B, Zhang J, Liu Y, Shi P, Wei G (2018). Co-occurrence patterns of soybean rhizosphere microbiome at a continental scale. Soil Biol. Biochem..

[52] Wiens JJ (2010). Niche conservatism as an emerging principle in ecology and conservation biology. Ecol. Lett..

[53] Deng Y (2012). Molecular ecological network analyses. BMC Bioinf..

[54] Liao H (2020). Complexity of bacterial and fungal network increases with soil aggregate size in an agricultural Inceptisol. Appl. Soil Ecol..

[55] Herren CM, McMahon KD (2018). Keystone taxa predict compositional change in microbial communities. Environ. Microbiol..

[56] Zhang C, Jiao S, Shu D, Wei G (2021). Inter-phylum negative interactions affect soil bacterial community dynamics and functions during soybean development under long-term nitrogen fertilization. Stress Biol..

[57] Su YG, Huang G, Lin YJ, Zhang YM (2016). No synergistic effects of water and nitrogen addition on soil microbial communities and soil respiration in a temperate desert. CATENA.

[58] Yang C (2020). Assessing the effect of soil salinization on soil microbial respiration and diversities under incubation conditions. Appl. Soil Ecol..

[59] Banerjee S (2016). Network analysis reveals functional redundancy and keystone taxa amongst bacterial and fungal communities during organic matter decomposition in an arable soil. Soil Biol. Biochem..

[60] Chen L-F, He Z-B, Zhao W-Z, Kong J-Q, Gao Y (2021). Empirical evidence for microbial regulation of soil respiration in alpine forests. Ecol. Indic..

[61] Liu S (2020). Decoupled diversity patterns in bacteria and fungi across continental forest ecosystems. Soil Biol. Biochem..

[62] Lynch MD, Neufeld JD (2015). Ecology and exploration of the rare biosphere. Nat. Rev. Microbiol..

[63] Chen L (2019). Competitive interaction with keystone taxa induced negative priming under biochar amendments. Microbiome.

[64] Chiba A (2021). Soil bacterial diversity is positively correlated with decomposition rates during early phases of maize litter decomposition. Microorganisms.

[65] Li S, Wang S, Fan M, Wu Y, Shangguan Z (2020). Interactions between biochar and nitrogen impact soil carbon mineralization and the microbial community. Soil Till. Res..

[66] Bao S (2000). Soil agrochemical analysis.

[67] Zhai L, Liu H, Zhang J, Huang J, Wang B (2011). Long-term application of organic manure and mineral fertilizer on N_2_O and CO_2_ emissions in a red soil from cultivated maize-wheat rotation in China. Agr. Sci. China.

[68] Xia W (2011). Autotrophic growth of nitrifying community in an agricultural soil. ISME J..

[69] Pruesse E (2007). SILVA: A comprehensive online resource for quality checked and aligned ribosomal RNA sequence data compatible with ARB. Nucleic Acids. Res..

[70] Benjamini Y, Hochberg Y (1995). Controlling the false discovery rate: a practical and powerful approach to multiple testing. J. R. Stat. Soc. B..

[71] Layeghifard M, Hwang DM, Guttman DS (2017). Disentangling interactions in the microbiome: A network perspective. Trends Microbiol..

[72] Liaw A, Wiener M (2002). Classification and regression by randomForest. R news.

[73] Archer E (2020). rfPermute: Estimate permutation p-values for random Forest importance metrics. R package version.

[74] Hooper D, Coughlan J, Mullen M (2008). Structural equation modelling: Guidelines for determining model fit. Electron. J. Bus. Res. Methods.

