# Supplementary material for: Fertilization treatments affect soil CO2 emission through regulating soil bacterial community composition in the semiarid Loess Plateau
Source: Sci Rep. 2022 Nov 22;12:20123. doi: 10.1038/s41598-022-21108-4 (PMC9684500; doi:10.1038/s41598-022-21108-4)
Supplement: Supplementary file 1 — Supplementary Information. [file 41598_2022_21108_MOESM1_ESM.docx]

**Fertilization treatments affect soil CO_2_ emission through regulating soil bacterial community composition in the semiarid Loess Plateau**

Jinbin Wang ^1,2^, Junhong Xie ^1,2^, Lingling Li ^1,2 *^, Zechariah Effah ^1,2^, Lihua Xie ^1,2^, Zhuzhu Luo ^1,3^, Yongjie Zhou ^1,2^, Yuji Jiang ^4 *^

^1^ State Key Laboratory of Aridland Crop Science, Gansu Agricultural University, Lanzhou, 730070, China

^2^ College of Agronomy, Gansu Agricultural University, Lanzhou, 730070, China

^3^ College of Resource and Environment, Gansu Agricultural University, Lanzhou, 730070, China

^4^ State Key Laboratory of Soil and Sustainable Agriculture, Institute of Soil Science, Chinese Academy of Sciences, Nanjing, 210008, China

Corresponding address: lill@gsau.edu.cn (Lingling Li); yjjiang@issas.ac.cn (Yuji Jiang)

**Supplementary Table S1–S3**

**Table S1.** The relationships between bacterial dominant phyla and CO_2_ emission.

**Table S2.** Topological properties of the bacterial network.

**Table S3.** The keystone taxa in the bacterial networks under different treatments.

**Supplementary Figures S1–S3**

**Figure S1.** Abundance and alpha-diversity of soil bacterial communities under different treatments.

**Figure S2.** The structure of soil bacterial communities by principal coordinate analysis (PCoA), which is constrained by four treatments based on Bray-Curtis distances.

**Figure S3.** Associations between C emission and relative abundances of keystone taxa across all treatments.

**Table S1.** The relationships between bacterial dominant phyla and CO_2_ emission.

| Dominant phyla | *r* | *p* |
| --- | --- | --- |
| Alphaproteobacteria | 0.759 | 0.001 |
| Gammaproteobacteria | 0.764 | 0.001 |
| Acidobacteriota | -0.84 | 0.000 |
| Actinobacteriota | -0.205 | 0.465 |

**Table S2.** Topological properties of the bacterial network.

| Network indexes | Value |
| --- | --- |
| Nodes | 140 |
| Edges | 798 |
| ACC | 0.475 |
| APL | 2.857 |
| Diameter | 7 |
| Density | 0.082 |
| Positive edges | 750 |
| Negative edges | 48 |

ACC, average clustering coefficients; APL, average path length.

**Table S3.** The keystone taxa in the bacterial networks.

| ID | Phylum | Family | Degree | Eigen_  centrality | Closeness  centrality | Betweenness  centrality |
| --- | --- | --- | --- | --- | --- | --- |
| OTU21 | Gammaproteobacteria | Nitrosomonadaceae | 25 | 0.772 | 0.421 | 263.1663 |
| OTU44 | Chloroflexi | norank | 27 | 0.88 | 0.416 | 285.0826 |
| OTU58 | Acidobacteriota | Vicinamibacteraceae | 30 | 0.97 | 0.436 | 252.8851 |
| OTU77 | Actinobacteriota | norank | 29 | 0.92 | 0.424 | 214.9271 |
| OTU92 | Myxococcota | norank | 28 | 0.88 | 0.433 | 299.4519 |
| OTU177 | Alphaproteobacteria | Beijerinckiaceae | 28 | 0.81 | 0.425 | 367.8697 |


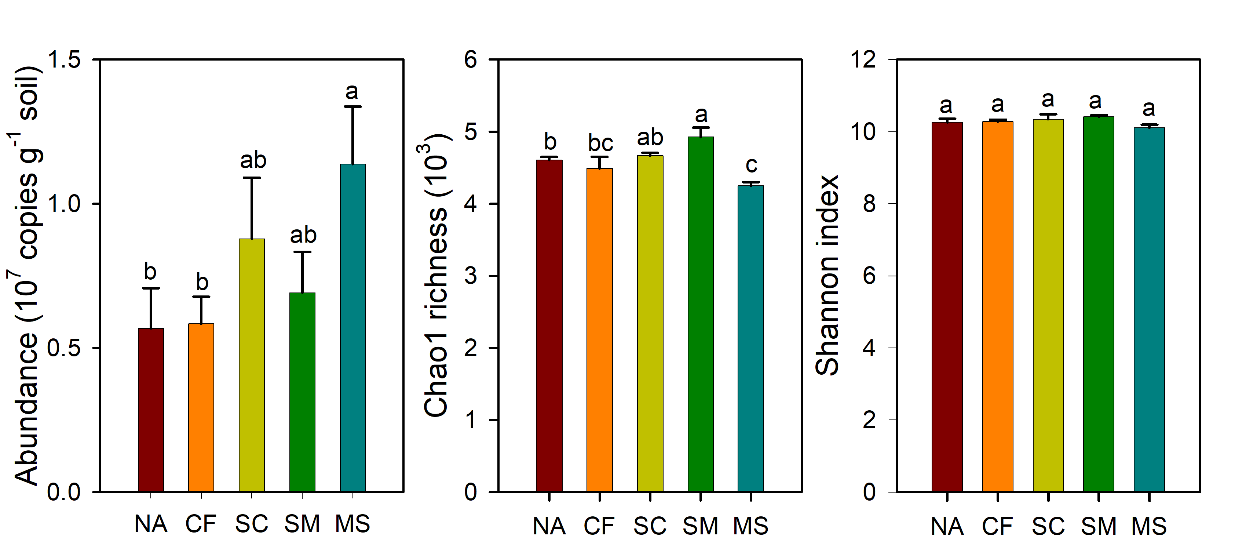


**Figure S1.** Abundance and alpha-diversity of soil bacterial community under different treatments. NA, no fertilizer; CF, inorganic fertilizer; SC, inorganic fertilizer plus organic fertilizer; SM, organic fertilizer; MS, maize straw. Different letters indicate significant difference at *P* < 0.05.


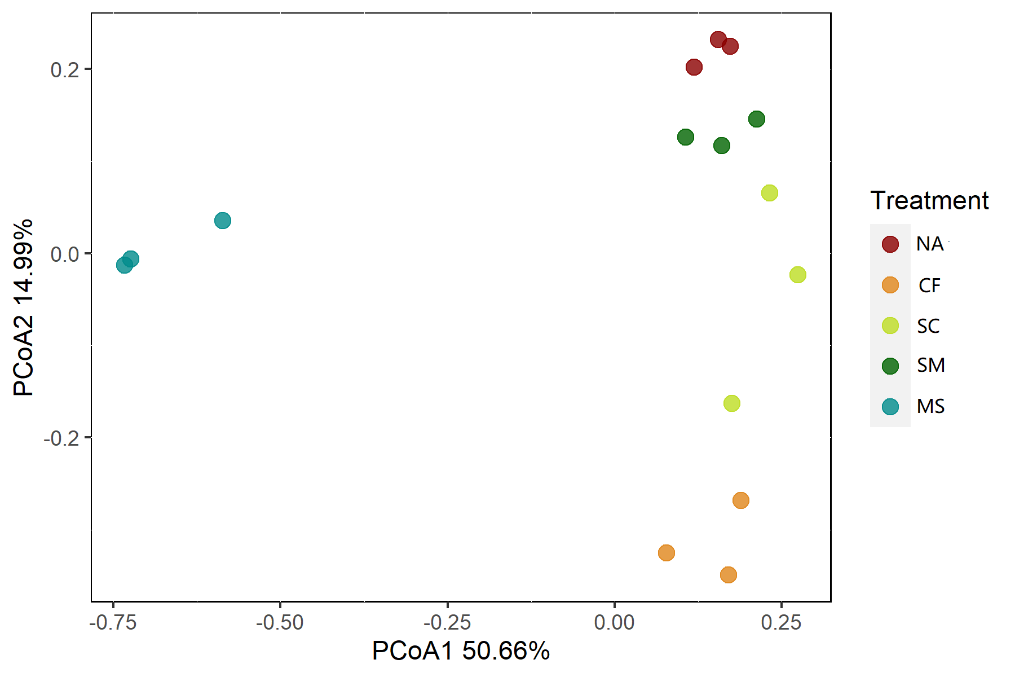


**Figure S2.** The structure of soil bacterial community by principal coordinate analysis (PCoA), which is constrained by four treatments based on Bray-Curtis distances. NA, no fertilizer; CF, inorganic fertilizer; SC, inorganic fertilizer plus organic fertilizer; SM, organic fertilizer; MS, maize straw.

**
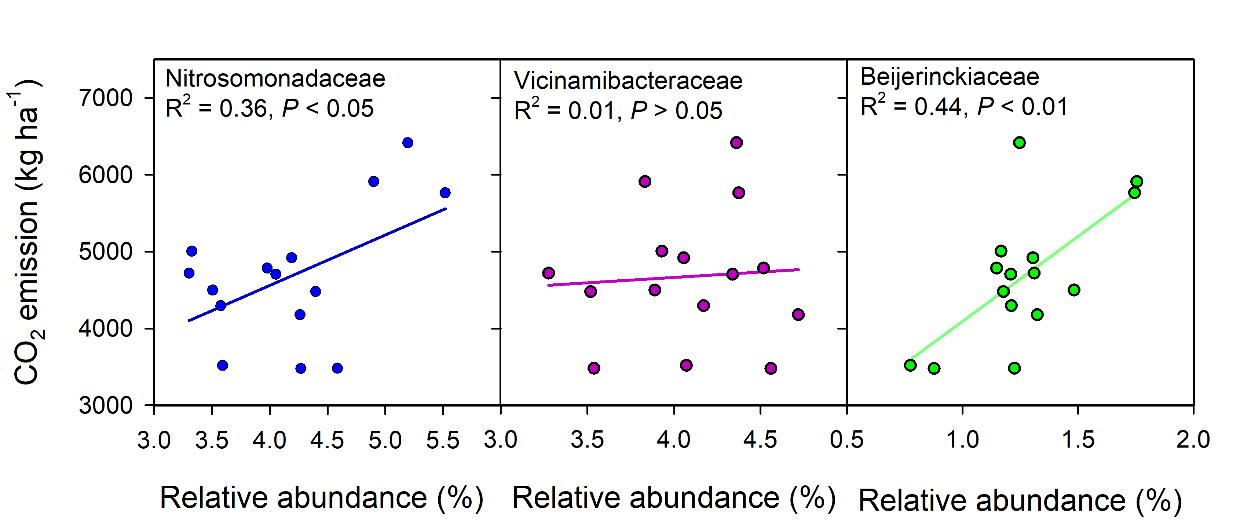
**

**Figure S3.** Associations between CO_2_ emission and relative abundances of keystone taxa across all treatments.
